# Anterolateral Ligament Expert Group consensus paper on the management of internal rotation and instability of the anterior cruciate ligament - deficient knee

**DOI:** 10.1007/s10195-017-0449-8

**Published:** 2017-02-20

**Authors:** Bertrand Sonnery-Cottet, Matthew Daggett, Jean-Marie Fayard, Andrea Ferretti, Camilo Partezani Helito, Martin Lind, Edoardo Monaco, Vitor Barion Castro de Pádua, Mathieu Thaunat, Adrian Wilson, Stefano Zaffagnini, Jacco Zijl, Steven Claes

**Affiliations:** 1 Centre Orthopédique Paul Santy, FIFA Medical Center of Excellence, Groupe Ramsay-Generale de Santé, 24 avenue Paul Santy, 69008 Lyon, France; 2 Kansas City University, Kansas City, MO USA; 3 Orthopaedic Unit and Kirk Kilgour Sports Injury Center, Sant’Andrea University Hospital, “Sapienza” University of Rome, Rome, Italy; 4 Knee Surgery Division, University of São Paulo, São Paulo, Brazil; 5 Division of Sportstraumatology, Department of Orthopedics, Aarhus University Hospital, Tage Hansens Gade 2, 8000 Aarhus C, Denmark; 6 Associação Beneficente Hospital Universitário de Marilia-SP Brazil, Cidade Universitária, Rua Dr Prospero Cecilio Coimbra 80, Marilia, SP 17525-160 Brazil; 7 Department of Sport and Exercise, Sport and Exercise Research Centre, University of Winchester, Winchester, SO22 4NR UK; 8 Dipartimento Rizzoli Sicilia, Ortopedia e Traumatologia, Università di Bologna, SS 113 al km 246, 90011 Bagheria, PA Italy; 9 St. Antonius Hospital, Soestwetering 1, 3543 AZ Utrecht, The Netherlands; 10 Department of Orthopaedic Surgery and Traumatology, AZ Herentals, Herentals, Belgium

**Keywords:** Anterolateral ligament, Anterolateral ligament reconstruction, Anterior cruciate ligament, Pivot-shift, Segond fracture

## Abstract

Purpose of this paper is to provide an overview of the latest research on the anterolateral ligament (ALL) and present the consensus of the ALL Expert Group on the anatomy, radiographic landmarks, biomechanics, clinical and radiographic diagnosis, lesion classification, surgical technique and clinical outcomes. A consensus on controversial subjects surrounding the ALL and anterolateral knee instability has been established based on the opinion of experts, the latest publications on the subject and an exchange of experiences during the ALL Experts Meeting (November 2015, Lyon, France). The ALL is found deep to the iliotibial band. The femoral origin is just posterior and proximal to the lateral epicondyle; the tibial attachment is 21.6 mm posterior to Gerdy’s tubercle and 4–10 mm below the tibial joint line. On a lateral radiographic view the femoral origin is located in the postero-inferior quadrant and the tibial attachment is close to the centre of the proximal tibial plateau. Favourable isometry of an ALL reconstruction is seen when the femoral position is proximal and posterior to the lateral epicondyle, with the ALL being tight upon extension and lax upon flexion. The ALL can be visualised on ultrasound, or on T2-weighted coronal MRI scans with proton density fat-suppressed evaluation. The ALL injury is associated with a Segond fracture, and often occurs in conjunction with acute anterior cruciate ligament (ACL) injury. Recognition and repair of the ALL lesions should be considered to improve the control of rotational stability provided by ACL reconstruction. For high-risk patients, a combined ACL and ALL reconstruction improves rotational control and reduces the rate of re-rupture, without increased postoperative complication rates compared to ACL-only reconstruction. In conclusion this paper provides a contemporary consensus on all studied features of the ALL. The findings warrant future research in order to further test these early observations, with the ultimate goal of improving the long-term outcomes of ACL-injured patients.

*Level of evidence* Level V—Expert opinion.

## Introduction

After Steven Claes had authored the re-discovery paper about the anterolateral ligament (ALL) of the knee in 2013, this “new” anatomical structure was cast into the spotlight by the lay media [15]. Since this date, orthopaedic surgeons have demonstrated a renewed interest in the anterolateral structures of the knee, with more than 85 articles being published on the anterolateral ligament.

Despite this extensive research effort, there is no consensus whether or not the ALL exists and which functions it serves; on the contrary, the ALL is a highly controversial subject. For some authors this anatomical structure either does not exist or has no function in knee stability [38, 44, 60, 76]. For others authors, its existence has been demonstrated macroscopically in all knees, and its histologic appearance has been identified as a ligamentous structure [9, 19, 32, 102]. Furthermore, the ALL appears to be involved in the rotational control of the knee [71, 87].

A very similar controversy concerning the medial patellofemoral ligament (MPFL) plagued the field of knee surgery after publication of the first clinical report on MPFL reconstruction in 1992 [24]. At that time, the existence and the function of the MPFL was heavily debated and challenged by many authors. The controversy was mainly due to the difficulty experienced in isolating the MPFL using different dissection protocols, and in identifying this structure by imaging [including magnetic resonance imaging (MRI)]. Nowadays, discussions around the MPFL mainly focus on the surgical indications for MPFL reconstruction and the surgical technique applied; its clinical role in patellar instability is now widely accepted by the orthopaedic community [84].

Further similarities can be found between the MPFL and the ALL. From an anatomical perspective, it appears that different ALL surgical dissection techniques have led to different rates of identification, as well as varying reports about ALL shape, location and dimension. Biomechanical function has been reported to be different, and different reconstruction techniques have been proposed. This high variability is not surprising if different structures, all being called the ALL, have been investigated [76].

It took many years for MPFL reconstruction to be widely adopted by orthopaedic surgeons. The ALL could be the anatomical missing link justifying the historical “lateral extra articular tenodesis” (e.g. Lemaire and MacIntosh procedures) [46, 52] for rotatory instability in anterior cruciate ligament (ACL) deficient knees. The ALL reconstruction procedure is still in an early phase of development and it is too soon to know if this procedure will be widely disseminated or not.

The goal of this consensus article is to update the orthopaedic community with the latest scientific knowledge on the ALL including: the history, anatomy, biomechanics, clinical diagnosis, classification of acute lesions, imaging, surgical indications, surgical techniques, post-operative protocol and clinical outcomes, based on recent publications and on the opinion of the ALL Expert Group.

## History

Interest in the anatomy and function of secondary restraints of the anterior cruciate ligament (ACL) has recently piqued among knee surgeons and researchers, as it is postulated that these structures/restraints play a very important role in both rotatory instability and the pivot-shift phenomenon in the ACL-deficient knee. Recently, Claes et al. [15] identified the ALL in an anatomic study as a distinct structure of the lateral compartment of the knee. This report, though heralded as the first clear identification of the ALL, is however preceded with reports of similar observations in the literature.

Historically, the first observations of the ALL can be accredited to the French anatomist Paul Segond. In 1897 he reported on a “pearly fibrous resistant band” showing extreme amounts of tension under excessive internal rotation, eventually resulting in an avulsion fracture as a result of a severe rotational stress [78]. Later examination revealed the “Segond fracture” to be an indirect sign of an ACL tear [22, 105]. It wasn’t until almost a century later that the ALL was once again described. In 1976 Hughston et al. described the “middle third of the lateral capsular ligament” as “technically strong”, and as a “major lateral static support at around 30° of flexion”, attaching proximally to the lateral epicondyle of the femur and distally at the tibial joint margin [37]. Tears of this structure resulted in an anterolateral rotatory instability, which could be revealed by a “jerk test”. Subsequently, in 1982, Müller [57] reported on the anatomy of the “anterolateral femorotibial ligament” as a distal, posterior portion of the iliotibial tract extending from the linea aspera of the femur to the Gerdy’s tubercle. He described this ligament as providing passive rotational stabilization of the knee. Müller also described injury to this structure in the context of an acute ACL tear and suggested that the structure could undergo surgical repair. Later, in 1988, Feagin [25] confirmed the findings of Hughston and Müller. He identified that the ALL is responsible for the avulsion of the tibial plateau in ACL tears, providing the anatomical explanation for the Segond fracture first observed a century previously. Following this, in 1993, Terry et al. [94]. reported on the capsulo-osseus layer of the iliotibial tract acting as an anterolateral ligament of the knee, and described its role together with the ACL as an inverted U (horseshoe) structure around the posterior aspect of the lateral femoral condyle. Aside from these main discoveries, there are a number of other authors who should also be credited for describing the ALL and for additionally postulating its importance in supporting the ACL to control rotational stability, including Irvine et al. [39], Puddu et al. [69], Campos et al. [7], Viera et al. [101], and Vincent et al. [102].

Despite these historical reports of the ALL, the most accurate anatomical description of the ALL has been provided by Claes et al. [15], and its importance with regard to knee stability has been confirmed in numerous biomechanical studies [42, 53, 66, 71].

## Clinical anatomy of the ALL

Further to the anatomical study of the ALL by Claes et al. [15]. the anatomical characteristics of the ALL have been investigated by numerous authors [19, 23, 32, 50, 102]. In order to accurately identify the ALL, the dissection technique described by Dagget can be used [18]. Thus far, the ALL has been consistently identified in nearly all specimens investigated [15, 19, 23, 32, 50, 102]. The various descriptions of the ALL have led to some debate regarding the exact specifications of the ligament; however, there is a consensus that the ALL is a triangular, anterolateral structure found deep to the iliotibial band (ITB).

**Fig. 1:**
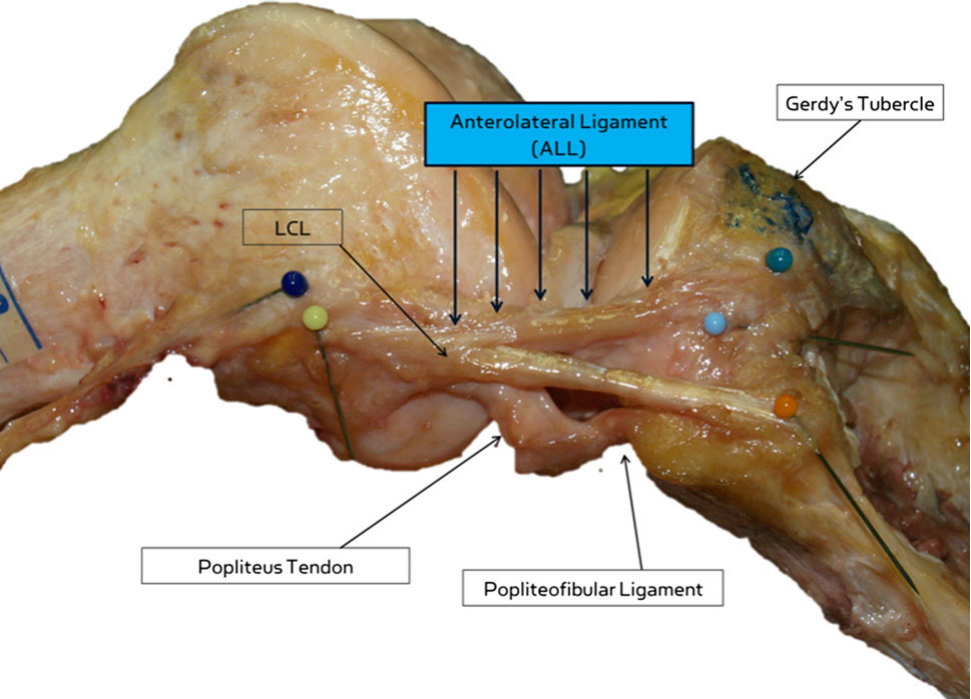
Anatomic dissection. The relationship of the anterolateral ligament (ALL) with the lateral collateral ligament (LCL), Gerdy’s tubercle, popliteofibular ligament and popliteus tendon From [15] by Anatomical Society. Reprinted with permission

According to Daggett [18], the ALL can be identified on cadaveric dissection (Fig. 1) by first carefully reflecting the ITB until its insertion at Gerdy’s tubercle. The biceps femoris is then reflected and the posterior and anterior margins of the ALL are identified with and internal rotational torque placed upon the tibia [18]. Key to successful identification of the ALL includes cautious dissection and separation of the ITB from the deeper structures, isolation of the biceps femoris, and combined flexion and rotation of the knee to identify the fibres of the ligament [18].

Analysis of the anatomy of the ALL in numerous specimens has revealed a certain amount of variability of the structure. The femoral origin of the ALL appears to vary [9, 15, 19, 23, 32, 50, 102] but is typically found just posterior and proximal to the lateral epicondyle [19]. The femoral origin directly adheres to the bone and has a mean diameter of 11.85 mm [19]. The ALL runs distally, immediately overlapping the proximal portion of the lateral collateral ligament [15]. As it approaches the joint line, some fibres of the ligament are attached to the lateral meniscus [31, 32] and the anterolateral capsule; [50] however, the majority of the fibres continue to run distally in a fan-like fashion, with the distal insertion being at the proximal tibia just behind Gerdy’s tubercle. The tibial attachment is 11.7 mm wide [9] and is centred 21.6 mm posterior to Gerdy’s tubercle [15], and 4–10 mm from the joint line [9, 15, 23, 32, 102].

The length of the ALL is between 34 mm [102] and 59 mm [23] from its femoral attachment to distal insertion. The thickness of the ALL also varies, and is particular in that it is nearly twice the thickness in males compared with females [18]. At a point just superior to the lateral meniscus at the level of the joint line, the thickness has been measured as 2.09 mm in males and 1.09 mm in females [18].

The consensus of the ALL Expert Group is as follows:

- The ALL is a distinct ligament at the anterolateral side of the human knee,
- The femoral attachment is posterior and proximal to the lateral epicondyle,
- The tibial attachment lies between Gerdy’s tubercle and the fibular head,
- the ALL has a constant attachment to the lateral meniscus.

## Radiographic anatomy

In order to optimise eventual treatment procedures, the radiographic anatomy of the ALL and its relationship with surrounding structures becomes a point of interest. With knowledge of the radiographic landmarks, fluoroscopy is known to effectively assist in graft positioning [41, 77]. For instance, this technique has been shown to be a successful method for tunnel positioning in contemporary MPFL reconstructions [77] and lateral collateral ligament reconstructions [41]. Accurate identification of the radiographic landmarks allows not only for minimally invasive reconstruction surgery, but also for a reconstruction which closely mimics the patient’s natural anatomy [41, 77].

With regard to the ALL, there are four published studies that focus on its radiographic landmarks [28, 33, 42, 72]. These studies reveal differences in the femoral landmark and similarities in the tibial landmark.

### Femoral origin

On a lateral view, Helito et al. [33]. used Blumensaat’s line as a reference point and identified the femoral attachment at approximately half way (47%) along Blumensaat’s line from the anterior edge of the femoral condyle [33]. Kennedy et al. used superimposed reference lines to establish femoral quadrants [42]. The first line was a parallel extension of the posterior femoral cortex. The second line was drawn perpendicularly to the posterior cortex extension and intersecting the most posterior aspect of Blumensaat’s line. The femoral attachment was identified in the postero-inferior quadrant, 8.4 mm proximal and posterior to the lateral epicondyle centre. Rezansoff et al. [72]. described the ALL origin as being along the posterior femoral cortical line, positioned between Blumensaat’s line and a line taken from the posterior condylar articular edge parallel to Blumensaat’s line. Heckmann et al. located the ALL origin at a distance of around 37% from the posterior edge of the femoral condyle, measured along Blumensaat’s line [28].

Anatomic variation in the ALL femoral attachment has been previously described by Daggett et al. and Helito et al. [19, 30]. To a certain degree, the variability in the identification of the femoral attachment may be due to differences in the dissection technique used. If the ALL origin is considered to be proximal and posterior to the lateral epicondyle, then it is possible that the radiographic landmark found by Kennedy et al. is accurate [42]. If the ALL origin is considered to be closer to the centre of the lateral epicondyle, then the landmark found by Helito et al. can be considered accurate [30].

### Tibial insertion

**Fig. 2:**
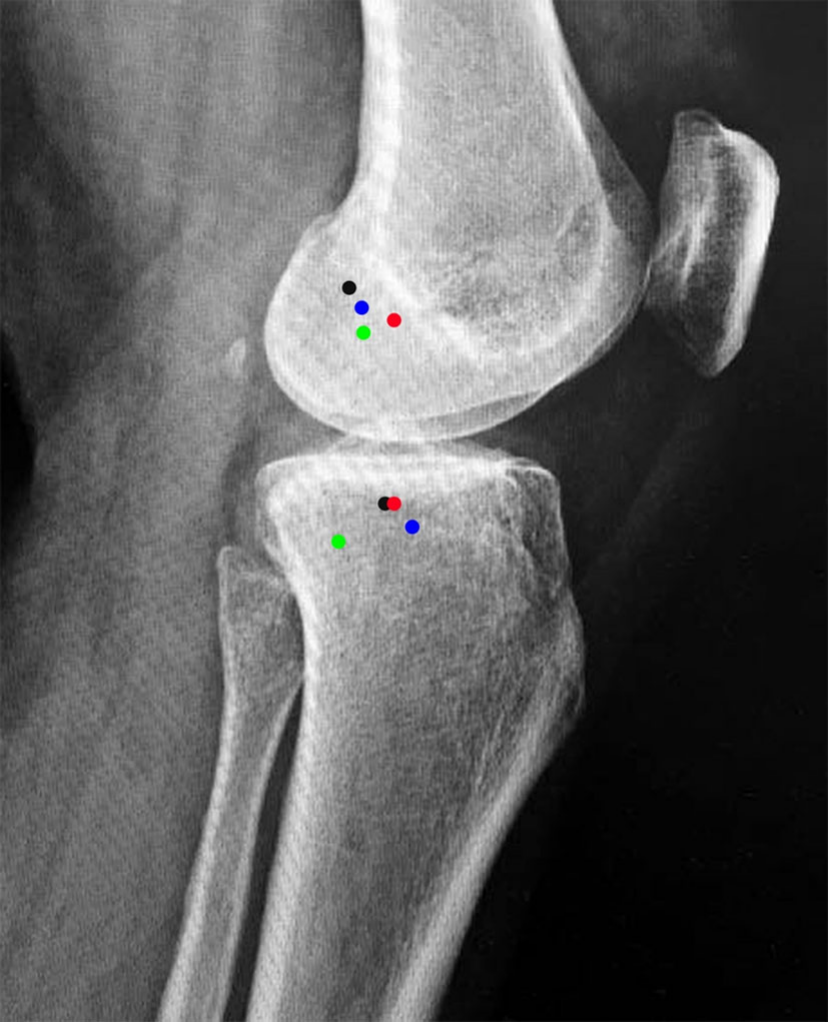
Radiographic landmarks. Lateral knee radiograph approximately showing the landmarks described by Helito et al. (*red*), Kennedy et al. (*black*), Rezansoff et al. (*green*) and Heckmann et al. (*blue*) [28, 32, 42, 72] (color figure online)

On a lateral view, the tibial landmark was found slightly posterior to the centre of the tibial plateau width by Helito et al. [33] and Kennedy et al. [32, 42] and slightly anterior to the centre of the tibial plateau width by Heckmann et al. [28]. However, Rezansoff et al. [72], described the tibial attachment as more posterior to the location identified by the other authors (Fig. 2).

On a frontal view, all authors identified the femoral landmark between 15.8 mm and 22.3 mm from the proximal joint line and the tibial attachment around 7 mm below the lateral tibial plateau [28, 33, 42, 72].

The consensus of the ALL Expert Group is as follows:

*Femoral origin*: on the lateral view, the femoral attachment is located in the postero-inferior quadrant described by Kennedy et al. [42]. On the frontal view, the femoral attachment is located 15 20 mm above the joint line.

*Tibial insertion*: on the lateral view, the tibial attachment is located close to the centre of the proximal tibial plateau. On the frontal view, the tibial attachment is located approximately 7 mm below the tibial joint line.

## Biomechanics

The ALL has been placed under the scientific microscope to closely examine its associated biomechanics which range from native structural properties to native and reconstructed kinematics. These studies focus in on the ALL, while not losing sight of surrounding lateral structures and the ACL. The reason for this close examination stems from the common goal of utilising an ALL reconstruction in the setting of an ACL deficiency, which thereby may eliminate residual rotational knee laxity and reduce the risk of ACL graft rupture in select patients. These patients may include ACL revision cases, the clinical presentation of joint hyperlaxity, and those with either high-demand for pivoting sports and/or presenting with a high grade pivot-shift diagnosis. Due to all of these recent studies, a consensus is now defined on what the ALL is and what role it plays in overall lateral knee stability. Furthermore, this information has provided the foundation to build effective and reproducible ALL reconstructions in combination with the treatment of a torn ACL.

Structural property tensile testing of the isolated ALL utilising similar specimen setup and crosshead speed (20 mm/min) has produced mean ultimate load values of 189 Newtons (N) and stiffness of 31 N/mm, when averaging the values of all 29 unpaired specimens [33, 46]. This structural data provides the rationale to select the appropriate autografts in conjunction with adequate fixation methods for reconstruction of the ALL.

In vitro robotic assessments of the ALL in the setting of an ACL injury have defined the ALL as a significant lateral knee stabiliser [74]. Specifically, the ALL has been demonstrated to act as a secondary stabiliser during internal rotation torque and simulated pivot-shift test in the ACL-deficient state. These results were further confirmed by other investigators utilising a surgical navigation system [90]. Within the discussion of these two papers, it became clear that a reconstruction of the ALL in conjunction with a torn ACL should be met with critical data, as the significant biomechanical importance lends itself to the need for sufficient and reproducible surgical techniques. Key points in this surgical treatment would involve techniques that provide stability without overconstraint while maintaining a minimally invasive, yet reproducible, surgical approach for this secondary stabiliser.

This was scientifically tested in part two of the in vitro robotic assessment with special attention to a combined reconstruction of the ALL and ACL [65]. In this study, the ALL reconstruction was able to further reduce the knee laxity when tested in conjunction with an ACL reconstruction. A primary finding was that during a simulated pivot-shift test, a significant reduction in internal rotation at 30°, 45° and 60° of knee flexion was noted for the ACL reconstruction in conjunction with an ALL reconstruction. This was statistically significant when compared to the ACL reconstruction with deficient ALL testing state. Favourable isometry is seen at the proximal and posterior to epicondyle femoral position, with the ALL being tight in extension and in internal rotation at 20° and lax at flexion at 120° and internal rotation at 90° [41]. These characteristics are of clinical importance, enabling optimisation of the femoral location in an ALL reconstruction.

The consensus of the ALL Expert Group is as follows:

Mean load to failure: around 180 N,

Mean stiffness: 31 N/mm,

Function: the ALL acts as a restraint for internal rotation of the tibia and affects the pivot-shift in the ACL-deficient knee.

## Clinical diagnosis

Diagnosing ALL lesions can be challenging even for expert clinicians. To date, no clinical tests have been validated for the diagnosis of ALL injuries. An appropriate diagnosis can only be obtained with a detailed anamnesis describing the mechanism of trauma, a meticulous clinical examination and appropriate evaluation of the radiographic and MRI imaging.

Generally, a combined ACL-ALL lesion occurs with trauma mechanisms similar to an isolated ACL injury. Contact and non-contact injuries involving early flexion, dynamic valgus and internal rotation, which occur during sport, are frequently reported [20, 88]. Considering that in severe cases an ALL injury is considered to represent a Segond fracture [14, 42], it is clear that symptoms related to a Segond fracture may be present during the acute phase of injury. Symptoms include elicited pain on palpation of the lateral tibial profile, increased laxity in varus stress, and during the Drawer test with the foot in external rotation; during varus stress elevation of the anterolateral capsule due to detachment from the tibia can be observed.

On examination, the lateral compartment of the knee should be carefully evaluated. Any swelling with tenderness over the lateral aspect of the knee, particularly if proximal to the head of the fibula but distal to the lateral joint line, should be assessed. Furthermore, the integrity of the medial collateral, lateral collateral and posterior cruciate ligaments should be examined.

If patient compliance allows for a safe and effective evaluation of ACL and ALL integrity, antero-posterior and rotatory laxity tests can be performed in the acute phase. However, evaluation is more effective in the later subacute and chronic phases, after swelling and pain has subsided.

Anterior drawer and Lachman tests are usually positive, with either a soft endpoint or no endpoint due to the ACL injury. The biomechanical properties of the ALL allow for internal rotation to be increased to over 30° of flexion; [71, 87] however, the pivot-shift seems to represent the most reliable test to evaluate ALL integrity. Monaco et al. demonstrated that a grade III pivot shift is only seen in the absence of both the ACL and ALL in vitro [53]. This finding is supported by several other recent biomechanical studies [71, 87], which reported increased coupled internal rotation, and lateral tibia anterior displacement after ALL sectioning in ACL-deficient models. In the clinical setting, anterolateral capsule abnormalities are reported on MRI imaging in 20, 40 and 73% of patients with grade I, grade II and grade III pivot-shift, respectively [85]. Great attention to the status of the ALL should thus be given in the evaluation of rotatory laxity in the ACL-deficient knee. Furthermore, as the pivot-shift has been reported to be influenced by high inter-examiner variability, standardisation of the test is recommended. Despite this, care should be taken to consider the potential confounding factors of a high-grade pivot-shift, such as a deficient lateral meniscus or root tear [59, 80], lateral posterior tibial slope >10.6° [85], ITB injury, or general hyperlaxity [91].

The consensus of the ALL Expert Group is that the pivot-shift test should be executed as follows: [59].

*Step 1* The examiner should control the patient’s slightly abducted leg with the ipsilateral hand placed at the heel level, imparting an internal rotation.

*Step 2* The contralateral hand should be placed on the lateral side of the joint with the thumb positioned just below the level of the proximal tibia-fibula joint. Gentle valgus stress should be applied. The knee should be naturally flexed with the combined stress of internal rotation and valgus stress.

**Fig. 3:**
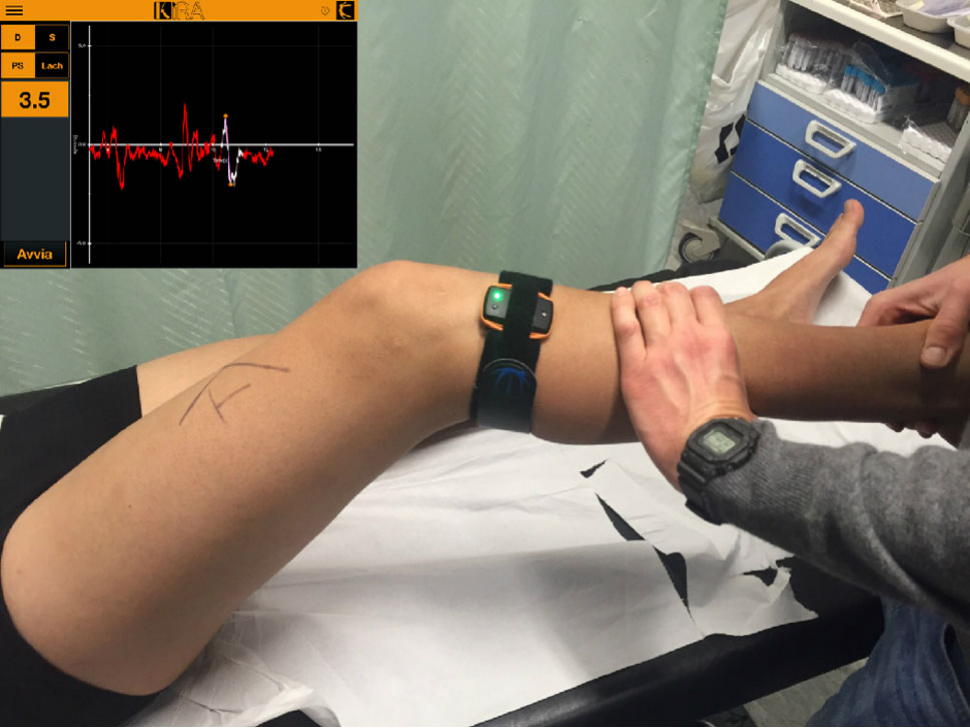
Quantification of the pivot-shift. KiRa (Orthokey LLC, DE, USA), a triaxial accelerometer is used

*Step 3* Knee flexion should be advanced with both hands. Internal rotation and valgus stress should be maintained until approximately 20° of knee flexion (Fig. 3). At the point of shifting, the rotational stress of the ipsilateral hand should be released, and the proximal tibia should be guided into external rotation by the contralateral hand. Therefore, at the time of shifting, the lateral side of the proximal tibia will suddenly drop by gravity and the tension of the ITB.

The examiner should record the pathological motion elicited in the test as: grade 0—normal, grade I—glide pivot, grade II—a jerk with subluxation or clunk, and grade III—significant clunk with locking (impingement of the posterolateral tibial plateau against the femoral condyle). For this purpose, objective methods for quantitative evaluation of rotatory laxity, such as accelerometers [3, 106], image analysis, or electromagnetic devices [55] would contribute to a more accurate diagnosis of ALL injury, and could represent the future direction of clinical diagnosis of ALL injuries.

## Diagnostic imaging procedures

**Fig. 4:**
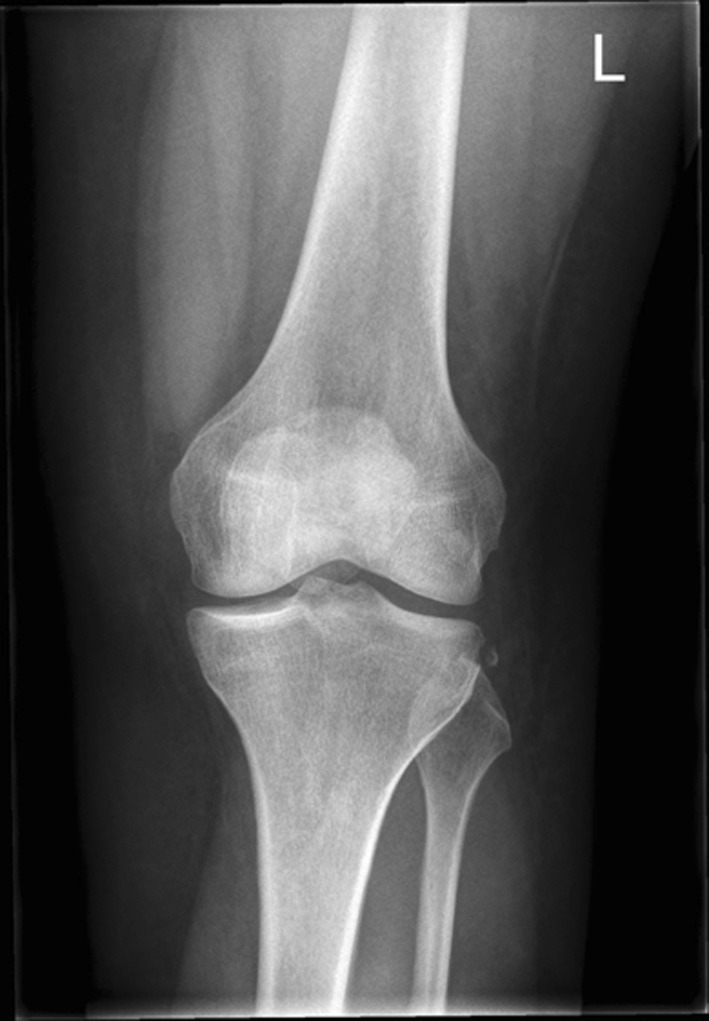
Anteroposterior X-ray indicating a Segond fracture

The clinical diagnosis of an ALL injury can be supported by radiographic imaging. Multiple imaging modalities have been reported to provide additional information on a possible injury of the ALL. Firstly, as previously described, a Segond fracture represents a bony injury of the tibial ALL insertion [14] (Fig. 4). A Segond fracture refers to avulsion of a cortical fragment of the tibia, posterior and proximal to Gerdy’s tubercle. A Segond fracture can be caused by high ALL tension forces, and is often the result of internal rotation of the knee and possibly varus stress [7, 9, 14, 42]. Such fractures can be visualised on straight, anteroposterior radiographs of the knee.

Secondly, the ALL can be visualised on routine, coronal MRI scans using T2-weighted sequences and proton density fat-suppressed evaluation. If the ALL is divided into a femoral, meniscal and tibial portion, it is the tibial attachment of the ALL which is most consistently seen on MRI scans [34, 92]. Two studies have shown that, on MRI scans, ALL abnormalities are frequently located in the distal part of the ligament, although some controversy still exists [13, 35, 98]. Despite this, ALL tears are difficult to consistently diagnose on standard 1.5T MRI sequences, and special sequences may be needed [27]. On MRI, one indication of ALL injury is the presence of bone marrow oedema as a result of a recent and violent pivot-shift trauma; in the acute post-traumatic phase, bone marrow oedema can be seen in the lateral femoral condyle and bilaterally on the posterior tibial plateau [21]. With the growing knowledge about the ALL, radiologists are becoming more used to its evaluation and protocols for evaluation are emerging [99].

Thirdly, ultrasound imaging can be of additional value in directly diagnosing ALL injury. Again, the tibial (distal) portion of the ligament is visualised better than the femoral portion [12], with the meniscal portion being difficult to identify. Since most ALL tears appear in the distal part, ultrasound may be a useful diagnostic tool to visualize ALL lesions [8, 65].

The consensus of the ALL Expert Group is as follows:

The intact ALL may therefore be identified using MRI or ultrasound techniques. However, reliable imaging evaluation of the injured ALL requires further research and collaboration with radiologists to develop more refined MRI protocols to aid in the detection of the ALL. Only then can imaging be reliably used in clinical examination and to support decisions for ALL treatment.

## Surgical indications

Persisting rotatory instability, indicated by a positive pivot shift, may be present in up to 25% of cases after an isolated intra-articular ACL reconstruction procedure; furthermore, persisting rotational instability has been shown to be a risk factor for recurrent injuries [10, 48]. Specific populations have a greater risk of persistent pivot shift and/or subsequent ipsilateral ACL tears. Improving the control of the rotational stability is mandatory for these patients. Female paediatric patients; [103] active patients who return to their preinjury level of activity [6]; elite athletes [67, 70] show a high rate of re-rupture and contralateral tears. Return to some specific activities including pivoting (e.g. skiing or volleyball) or contact sports (e.g. football or rugby) is also known to be a risk factor for ipsilateral and contralateral ACL rupture [1, 6, 70]. It is therefore important that the goals of a combined ACL and ALL reconstruction are to reduce the ACL graft re-rupture rate, and improve control of the rotational stability of the knee.

Any surgical indication is based on a favourable risk–benefit balance. Specific complications associated with more invasive additional extra-articular reconstruction have been reported [2, 54, 75]; although the principle might be the same, the proposed modern minimally invasive ALL reconstruction techniques differ significantly from these extra-articular reconstructions. The increasing knowledge about the ALL anatomy and function has allowed definition of the basis of this minimally invasive reconstruction, with an isometric positioning of the tunnels and a specific focus in the position of the fixation of the graft. A recent study evaluating minimally invasive ACL and ALL reconstructions demonstrated good short-term subjective and objective results without specific complications [88].

**Fig. 5:**
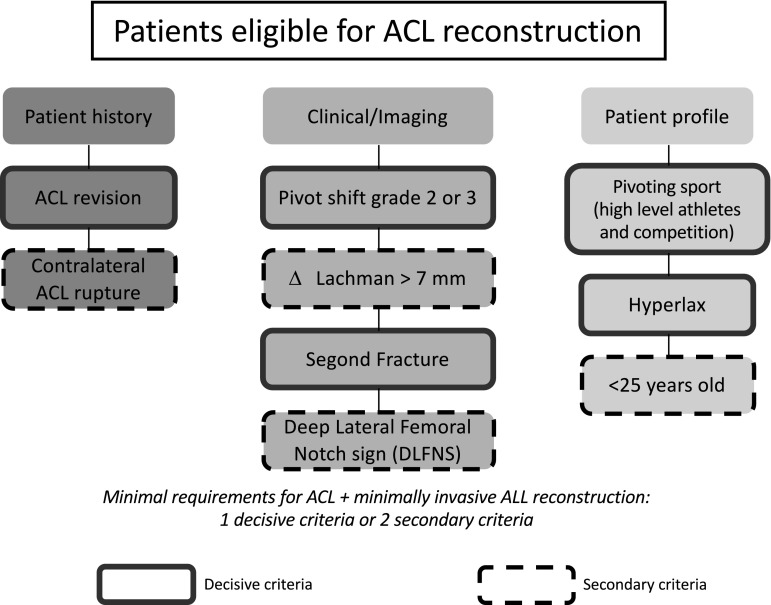
Decision tree

The ALL Expert Group consensus is that the minimally invasive ALL reconstruction is an extra-articular procedure that leads to similar postoperative outcomes and has a similar complication rate to the isolated ACL reconstruction. We propose a decision tree for the management of ACL ruptures (Fig. 5). A combined ACL and ALL reconstruction should be considered for patients who present at least:

- One decisive criteria for increased risk of secondary ACL rupture or postoperative residual positive pivot shift, or,
- Two secondary criteria for increased risk of secondary ACL rupture or postoperative residual positive pivot shift including history, clinical or imaging signs, or patient profile.

## Acute lesions

In order to support the diagnosis of ALL lesions, and aid in the decision to undertake surgical reconstruction, it is important to classify ALL lesions. Until now, the majority of studies on the ALL have focussed on anatomy [15] and biomechanics [53]. A recent study by Ferretti et al. [26]. investigated the prevalence and patterns of injuries of the lateral compartment in 60 patients with apparently isolated acute ACL tears, as diagnosed by clinical examination and confirmed by MRI. To evaluate potential concomitant ALL lesions, the lateral compartment was surgically exposed, the injuries were identified and subsequently recorded, photographed and repaired. Macroscopic tears of the lateral capsule were clearly identified in 90% of patients (54 patients).

The lesions were classified into four categories:

*Type I* Multilevel rupture in which individual layers are torn at different levels with macroscopic haemorrhage involving the ALL and extending to the anterolateral capsule only (19/60 patients, 31.6%).

*Type II* Multilevel rupture in which individual layers are torn at different levels with macroscopic haemorrhage extending from the ALL and anterolateral capsule to the posterolateral corner (16/60 patients, 26.7%).

**Fig. 6:**
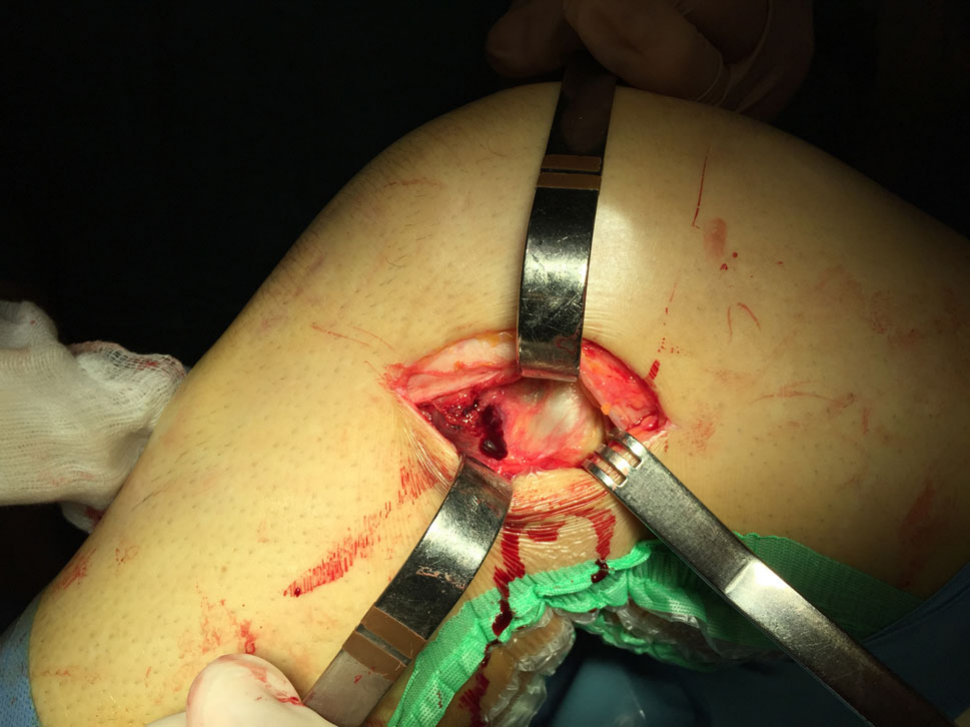
A type III lesion. The ALL and capsule near its insertion on the lateral tibial plateau are involved. Left knee

*Type III* Complete transverse tear involving ALL near its insertion into the lateral tibial plateau.

(13/60 patients, 21.7%) (Fig. 6).

*Type IV* Bony avulsion (a Segond fracture) (6/60 patients, 10%).

This study shows that injuries of the anterolateral secondary restraints often occur in cases of apparently isolated ACL tears. Moreover, they often involve a larger area of the lateral capsule extending beyond the ALL, known as the anterolateral complex. This study supports the previously described concept that rotational instability is a more complicated issue than simply the result of an ACL tear.

Historically, the location of injuries resulting in anterolateral rotational instability and their classification started in 1976, when Hughston et al. [37]. categorised three distinct portions of the lateral capsule-ligamentous tissues. Based on the evaluation of six patients undergoing surgery for acute anterolateral rotatory instability, the “mid-third lateral capsular ligament” was suggested to have an important role in anterolateral instability. Following this study, Norwood et al. [64] documented the presence of injuries of the lateral compartment in 36 knees with acute anterolateral rotatory instability, in which only three Segond fractures were diagnosed. Müller [57] later identified the anterolateral femorotibial ligament, which, in association with an ACL tear, was shown to exhibit visible avulsion from the femur or overstretching of the fibres. Later, Terry et al. [94] classified injuries of the lateral compartment in the presence of an ACL tear in a series of 82 cases of acute ACL injuries. In this study, 93% of injuries of the lateral compartment included transverse and interstitial superficial and deep layer tears.

The consensus of the ALL Expert Group is as follows:

As injuries of secondary restraints often occur in cases of acute ACL tears, recognition and repair of such lesions should be considered in order to improve the control of rotational stability provided by ACL reconstruction.

## ALL consensus group surgical technique

The ALL consensus group propose a surgical technique allowing for a minimally invasive and low morbidity procedure to recreate the ALL, which is crucial to anterolateral rotatory instability. The consensus opinion is to use a tendon graft with one limb attached to the femur at the correct anatomical position, and a single or double bundle (i.e. the so-called “delta”—or Y-construct) configuration at the tibia mimicking the native anatomy of the ALL. The ALL can be reconstructed in isolation, or more commonly in conjunction with an ACL reconstruction.

### Graft preparation

The preferred graft is the gracilis tendon. This is harvested in the standard way and both ends are whipstitched with a number 2 suture. The knee is flexed to 90° and held with a footrest and side support. The anatomy is identified and marked. The 3 key landmarks are the lateral epicondyle, the fibula head and Gerdy’s tubercle.

The femoral epicondyle is palpated and identified, preferentially before ACL femoral socket drilling. A 15 mm incision is made just proximal to the epicondyle and the ITB is divided. The lateral epicondyle is then palpated and a position taken 8 mm proximal and 4 mm posterior to the lateral epicondyle. A 2.4 mm drill pin is then inserted.

### Tibial socket identification

On the tibia the key anatomical landmarks are the centre of the fibula head and the centre of Gerdy’s tubercle. A stab incision is made 10 mm below the joint line, halfway between the centre of Gerdy’s tubercle and the fibula head, and a dissection is made to the bone. If a second tibial socket is planned, a second, more anterior incision is made over the centre of Gerdy’s tubercle using the delta-technique. A 2.4 mm wire is then placed through each incision in a tangential fashion to the tibial bone. A 4.5 mm cannulated drill bit is then used to create two bony sockets on the tibia.

### Isometry test

To ensure that the ALL graft will not tighten in flexion and will be functioning near extension, an isometry assessment is made. The passing suture is placed around the femoral wire and then in turn around each of the tibial wires, and the knee is taken through a full range of motion. The suture should be tighter in extension and become lax as the knee is taken into flexion. If the suture tightens in flexion, then the femoral socket position is too distal and anterior and should be adjusted accordingly.

### Femoral socket preparation and fixation

A 4.5 mm cannulated drill is used to create a socket to a depth of 20 mm to fully accommodate the bone anchor. The mouth of the tunnel is debrided and cleared to ensure easy passage of the graft. The gracilis graft is then placed into the femoral socket and the screw is advanced in the standard way.

### Graft passage and fixation

**Fig. 7:**
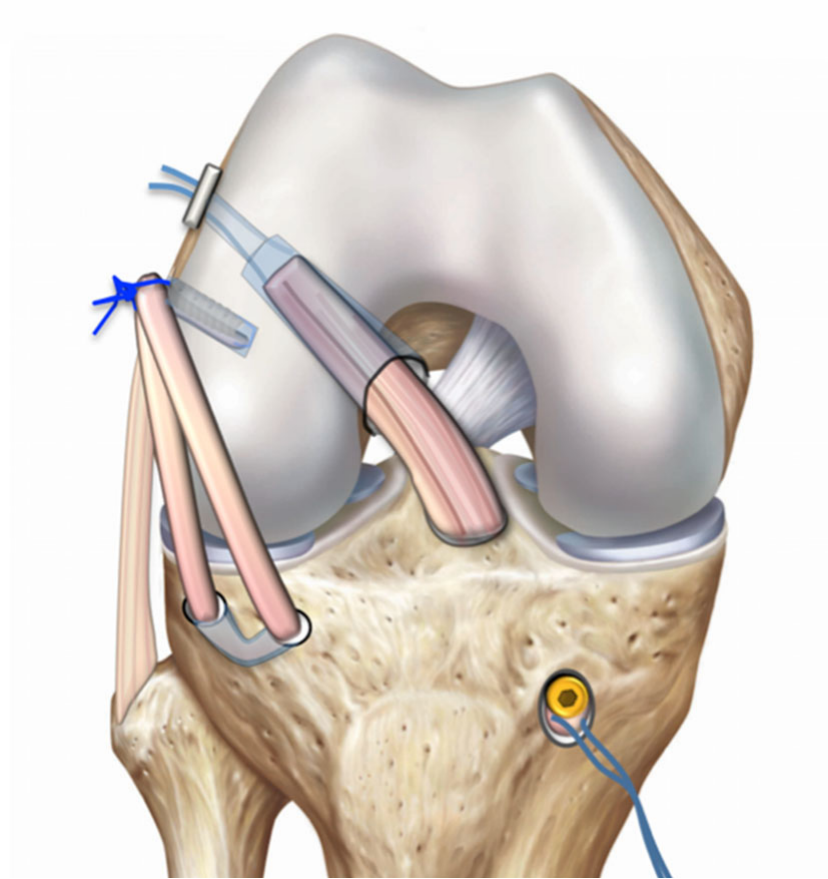
Surgical technique. Drawing depicting a schematic view following a combined ACL/ALL reconstruction

Blunt dissection is carried out under the ITB to make a communication between the femoral socket and the tibial socket(s). Final tensioning is then carried out. The knee is taken into full extension, which ensures the foot is in neutral rotation, and the graft is fixed in the tibial tunnel(s) with a 4.5 mm anchor (Fig. 7). The knee is then cycled through a full range of motion several times and a final check is made of the delta graft to ensure appropriate tension has been obtained. Local anaesthetic is infiltrated and the wounds are closed in layers.

## Post-operative protocol

The aim of ALL reconstruction is to reproduce the natural anatomy, which can be achieved through correct graft placement and fixation enabling efficient extra-articular reconstruction [45, 88]. This minimizes possible post-operative complications such as lateral constraint, loss of motion and graft failure [45, 58]. It is the recommendation of the ALL Consensus Group that rehabilitation after an ALL reconstruction, particularly if performed in conjunction with an ACL reconstruction, should be carried out in a similar way to conventional ACL rehabilitation [68].

An accelerated rehabilitation program can be offered [79]. This should comprise initial quadriceps awakening with both voluntary and electro stimulated muscle contraction, and emphasis should be placed on achieving immediate full extension to reduce the risk of bleeding and adhesion or cyclops formation. Passive flexion and patellar mobilization, avoiding eccentric quadriceps contraction, should also be performed. The patient can be discharged on the same day or on the day after surgery, without immobilisation, and should be total weight-bearing (as tolerated) with the aid of crutches.

The ALL Consensus Group rehabilitation protocol is a six stages protocol, described as follows:

### Stage 1 (up to 2 weeks)

- Quadriceps awakening with full extension,
- Control of inflammatory signs, pain and effusion,
- Gait training,
- Active and passive range of motion, at least 90°,
- Hamstrings stretching to prevent flexion attitude.

### Stage 2 (2–6 weeks)

- Normal walking without crutches (from the point of sufficient neuromuscular control without limping),
- Active range of motion,
- Isometric closed kinetic chain (0–50°) to avoid anterior tibia translation,
- Progressive muscle strengthening,
- Cycling on an ergometer,
- Body balance training,
- Hamstring strengthening: attention to pseudo-flexion contractures,
- All strengthening should be carried out without causing pain or effusion.

### Stage 3 (6–12 weeks)

- Restoration of neuromuscular control,
- No swelling or pain,
- Normal range of motion,
- Increased strength,
- Closed kinetic chain (0-50°), leg press exercises,
- Lunges and squats, both legs,
- Avoid valgus knee dropping, emphasis on hip muscles as abductors and external rotators [61],
- Stepping.

### Stage 4 (12 weeks to 5 months)

- Start running,
- Jump and change direction without hesitation,
- Full program of strength,
- Non-pivoting sports.

### Stage 5 (5–6 months)

- Full range of motion,
- Agility training during simulation of sport activity,
- Regaining dynamic joint stability [100].

### Stage 6 (from 6 months)

- Sport-specific training and “return-to-play” exercises.

A return to sport is not based on time alone, but also on restored muscle function, which is reflected in strength and jumping ability [90]. Isokinetics can be used to improve strength, with different hop tests (other than the one-leg hop test for distance) being available for assessment of functional performance [56, 73, 104].

The consensus of the ALL Expert Group is as follows:

If a combined ALL and ACL reconstruction is performed with correct positioning, the course of rehabilitation should be smooth. Rehabilitation should follow the standard ACL rehabilitation protocol, described above.

## Clinical outcomes

Clinical results are the best way to address the biomechanical controversy surrounding the anterolateral ligament. Regardless of the type of ACL graft used, most studies report a rate of residual pivot-shift of up to 15% [62], with the rate of graft rupture rising to 17% in a young and elite athletic population [40]. Lateral tenodesis combined with ACL reconstruction reduces pivot-shift, but results in no significant difference in clinical outcome [36]. To our knowledge, there has been only one study on the clinical outcomes of combined ACL and ALL reconstruction: a prospective case series with a two-year follow up (no control group) reported by Sonnery-Cottet et al. in 2015 [88]. Eighty-three combined ACL with ALL reconstructions were performed. A semitendinosus tendon graft was used for ACL and a gracilis tendon graft for the percutaneous double-strand ALL reconstruction, in order to replicate the triangular shape of the native ALL. The ALL was secured in full extension to ensure neutral rotation of the tibia. The mean follow-up period was 32.4 months (range 24–39 months). Pre-operatively 47 patients had grade 1, 23 patients grade 2, and 19 patients grade 3 pivot-shift test results. Post-operatively 76 patients had a negative pivot-shift and 7 patients had grade 1 pivot-shift test results. Interestingly, no complications related to the surgical technique were reported and only one patient had an ACL graft rupture one year after the ACL reconstruction, whereas six patients had a contralateral ACL rupture. Given the results of combined ACL and ALL reconstruction compared to traditional ACL reconstruction in regards to re-rupture rate, return to play and rotational stability, it was concluded that the ALL has an important function concomitant to the ACL. These findings have been confirmed by the clinical experience of more than 1000 cases performed at the study location since 2011. The excellent outcome in stability and function, the simplicity of the technique (increase in operative time does not exceed 15 min), the minimal cosmetic impact resulting from the percutaneous technique, and the low failure rate has led to a dramatic expansion of surgical indications at the study centre during the last five years. In this centre, this technique is now performed in more than 70% of ACL reconstructions.

The consensus of the ALL Expert Group is that this combined technique not only allows for better rotational control, which unfortunately cannot be demonstrated objectively, but serves primarily to reduce the rate of re-rupture among high risk patients defined as under 20 years of age, high level athletes of pivot-sports, and hyperlaxity patients. The authors feel that the concerns raised about a potential overconstraint of the knee are neutralised if anatomical reconstruction is performed and the graft is fixed in full extension and neutral rotation. More prospective, randomised studies are needed to confirm these findings.

## Anterolateral ligament and ACL revision reconstruction

The causes for failure of ACL reconstruction have been suggested to be mainly due to new trauma within the first year of a return to sports, improper tunnel placement, or peripheral instability [47, 97]. The reason for the high risk of re-injury on returning to sports is poorly understood, but factors such as impaired proprioception and insufficient normalisation of knee functional stability after ACL reconstruction have been suggested [16]. Several studies have demonstrated abnormal knee rotational stability after ACL injury, and that ACL reconstruction typically cannot recreate normal rotational stability [11].

An anatomical ACL reconstruction using the double bundle reconstruction technique has recently been advocated to improve rotational stability. A study using robotic rotation analysis has demonstrated improved dynamic rotational stability with a double bundle reconstruction [81]. However, another clinical study using 3D motion analysis did not find improved functional rotational stability [4]. Unfortunately, a number of randomised studies investigating double bundle ACL reconstruction outcomes have not been able to demonstrate consistent improvement of rotational stability by reducing pivot shift [95].

Creation of improved rotational stability with lateral extra-articular reconstruction or tenodesis is a reasonable strategy to improve knee biomechanical properties of internal tibial rotation after failed ACL reconstructions. A few studies have looked at the impact of supplemental lateral tenodesis on the outcome after ACL revision reconstruction. One multicentre study demonstrated a reduced incidence of a positive pivot shift, but overall International Knee Documentation Committee (IKDC)-evaluated knee stability did not improve as a result of lateral tenodesis in combination with ACL reconstruction [96]. A biomechanical study using intraoperative navigation during revision ACL reconstruction demonstrated that addition of a lateral tenodesis resulted in improved tibial rotational stability at high flexion, but had no influence on sagittal stability [17]. In patients with ACL graft failure, an ALL reconstruction or lateral tenodesis could be considered in cases of high grade pivot shift, hyperlaxity and the desire to return to rotational sport activities. The improved biomechanical control of rotation by the lateral reconstruction could protect the new ACL graft during risk activities and return to sports. So far, no clinical studies have demonstrated that ALL reconstruction can reduce failure rates after ACL revision nor improve subjective outcomes or function. Despite this, one randomized controlled study for ACL revision patients with 100 patients randomized to ACL revision with or without ALL reconstruction using allograft tendon tissue is ongoing at the time of writing (NCT02680821).

The consensus of the ALL Expert Group is as follows:

Some ACL reconstructions fail due to insufficient rotational control on return to sports. In patients with objective excessive instability after ACL reconstruction, failure of a supplemental reconstruction or tenodesis of anterolateral structures, can be considered when performing ACL revision reconstruction. However, more clinical data is needed to soundly support a significant benefit of such a strategy.

## Future directions and conclusions

The ALL has been the surrounded by controversy since its recent in-depth characterisation. In 1879 Paul Segond already mentioned the presence of a ligamentous structure at the anterolateral side of the knee “showing extreme amounts of tension during forced internal rotation” [78]. Nowadays, it is clear that the ALL is a distinct anatomical structure at the anterolateral aspect of the human knee that is present in the vast majority of the studied cadavers [5, 15, 19, 32, 51, 89]. Furthermore, emerging scientific evidence confirms Segond’s observations that the ALL indeed restrains internal rotation of the tibia, and thus affects the pivot-shift phenomenon in the ACL-injured knees [50, 53, 63, 71, 74, 93].

Although our knowledge of ALL anatomy, function, imaging and treatment is increasing, many questions still remain unanswered. Until now, just one study on the clinical outcomes of combined ACL and ALL reconstruction has been published. More studies with longer follow-up times are therefore needed to provide the compelling clinical evidence for the efficacy of concomitant ACL and ALL procedures. Currently, the precise clinical indication for these procedures is still unknown. Although it seems obvious to reconstruct the ALL in ACL-deficient knees with high-grade rotational instability, the potential for natural healing of the ALL has not been studied to date. Further delineation of the ideal patient profile, identifying those patients who could benefit from an additional ALL reconstruction, will definitely facilitate clinical decision-making.

Many surgical techniques have historically been proposed in the 1970’s and 1980’s to treat the so-called “anterolateral rotatory instability (ALRI)”, most often with variations on a ITB tenodesis-type of procedure involving the ITB [82]. Some laboratory results on various “modern” anatomic ALL reconstruction procedures may seem conflicting at first, and definitely, among the existing techniques [29, 43, 49, 83, 86], no one has been proven superior to others, but in order to compare ex vivo and in vivo outcomes of contemporary ALL reconstruction techniques, proper terminology should be used.

This paper primarily sought to provide a comprehensive consensus on the anatomy of the ALL amongst other features. According to the ALL Expert Group’s analysis, the ALL primarily attaches proximal and posterior to the lateral epicondyle on the femur. Thus, we suggest that all future studies adhere to this consensus on the anatomy of the ALL and otherwise clearly provide a detailed and precise anatomic description of the studied ligament, if different anatomy was observed. Furthermore, the use of confusing terminology as “anterolateral capsule”, “anterolateral complex”, “capsule-osseous layer of the ITB”, etc. should be avoided when explicitly the ALL is investigated in order to allow data integration into the growing body of knowledge on this interesting structure.

The authors want to stress that this consensus paper has just one single goal: to improve the outcome of our ACL-injured patients. As with every significant scientific progression, the more we learn from studying one subject, the more questions and issues seem to arise. This should however not be considered as a problem, but rather as a challenge. In fact, as long as the most exiting scientific ideas are tested with the highest quality in orthopaedic research, one will eventually be able to see the bigger picture in these enigmatic instability patterns of the human knee.

## Abbreviations

ACL: Anterior cruciate ligament
ALL: Anterolateral ligament
MPFL: Medial patellofemoral ligament
MRI: Magnetic resonance image
IKDC: International Knee Documentation Committee

## References

[1] Andernord D, Desai N, Bjornsson H, Ylander M, Karlsson J, Samuelsson K (2015). Patient predictors of early revision surgery after anterior cruciate ligament reconstruction: a cohort study of 16,930 patients with 2-year follow-up. Am J Sports Med.

[2] Anderson AF, Snyder RB, Lipscomb AB (2001). Anterior cruciate ligament reconstruction. A prospective randomized study of three surgical methods. Am J Sports Med.

[3] Berruto M, Uboldi F, Gala L, Marelli B, Albisetti W (2013). Is triaxial accelerometer reliable in the evaluation and grading of knee pivot-shift phenomenon?. Knee Surg Sports Traumatol Arthrosc Off J ESSKA.

[4] Bohn MB, Sorensen H, Petersen MK, Soballe K, Lind M (2015). Rotational laxity after anatomical ACL reconstruction measured by 3-D motion analysis: a prospective randomized clinical trial comparing anatomic and nonanatomic ACL reconstruction techniques. Knee Surg Sports Traumatol Arthrosc Off J ESSKA.

[5] Bonasia DE, D’Amelio A, Pellegrino P, Rosso F, Rossi R (2015). Anterolateral ligament of the knee: back to the future in anterior cruciate ligament reconstruction. Orthop Rev (Pavia).

[6] Bourke HE, Salmon LJ, Waller A, Patterson V, Pinczewski LA (2012). Survival of the anterior cruciate ligament graft and the contralateral ACL at a minimum of 15 years. Am J Sports Med.

[7] Campos JC, Chung CB, Lektrakul N, Pedowitz R, Trudell D, Yu J, Resnick D (2001). Pathogenesis of the Segond fracture: anatomic and MR imaging evidence of an iliotibial tract or anterior oblique band avulsion. Radiology.

[8] Capo J, Kaplan DJ, Fralinger DJ, Adler RS, Campbell KA, Jazrawi LM, Alaia MJ (2016). Ultrasonographic visualization and assessment of the anterolateral ligament. Knee Surg Sports Traumatol Arthrosc Off J ESSKA.

[9] Caterine S, Litchfield R, Johnson M, Chronik B, Getgood A (2015). A cadaveric study of the anterolateral ligament: re-introducing the lateral capsular ligament. Knee Surg Sports Traumatol Arthrosc Off J ESSKA.

[10] Chouliaras V, Ristanis S, Moraiti C, Stergiou N, Georgoulis AD (2007). Effectiveness of reconstruction of the anterior cruciate ligament with quadrupled hamstrings and bone-patellar tendon-bone autografts: an in vivo study comparing tibial internal-external rotation. Am J Sports Med.

[11] Chouliaras V, Ristanis S, Moraiti C, Tzimas V, Stergiou N, Georgoulis AD (2009). Anterior cruciate ligament reconstruction with a quadrupled hamstrings tendon autograft does not restore tibial rotation to normative levels during landing from a jump and subsequent pivoting. J Sports Med Phys Fit.

[12] Cianca J, John J, Pandit S, Chiou-Tan FY (2014). Musculoskeletal ultrasound imaging of the recently described anterolateral ligament of the knee. Am J Phys Med Rehabil Assoc Acad Physiatr.

[13] Claes S, Bartholomeeusen S, Bellemans J (2014). High prevalence of anterolateral ligament abnormalities in magnetic resonance images of anterior cruciate ligament-injured knees. Acta Orthop Belg.

[14] Claes S, Luyckx T, Vereecke E, Bellemans J (2014). The Segond fracture: a bony injury of the anterolateral ligament of the knee. Arthrosc J Arthrosc Related Surg Off Publ Arthrosc Assoc N Am Int Arthrosc Assoc.

[15] Claes S, Vereecke E, Maes M, Victor J, Verdonk P, Bellemans J (2013). Anatomy of the anterolateral ligament of the knee. J Anat.

[16] Colombet P, Jenny JY, Menetrey J, Plaweski S, Zaffagnini S (2012). Current concept in rotational laxity control and evaluation in ACL reconstruction. Orthop Traumatol Surgery Res OTSR.

[17] Colombet PD (2011). Navigated intra-articular ACL reconstruction with additional extra-articular tenodesis using the same hamstring graft. Knee Surg Sports Traumatol Arthrosc Off J ESSKA.

[18] Daggett M, Busch K, Sonnery-Cottet B (2016). Surgical dissection of the anterolateral ligament. Arthrosc Tech.

[19] Daggett M, Ockuly AC, Cullen M, Busch K, Lutz C, Imbert P, Sonnery-Cottet B (2016). Femoral origin of the anterolateral ligament: an anatomic analysis. Arthrosc J Arthrosc Related Surg Off Publ Arthrosc Assoc N Am Int Arthrosc Assoc.

[20] Davis DS, Post WR (1997). Segond fracture: lateral capsular ligament avulsion. J Orthop Sports Phys Ther.

[21] De Maeseneer M, Boulet C, Willekens I, Lenchik L, De Mey J, Cattrysse E, Shahabpour M (2015). Segond fracture: involvement of the iliotibial band, anterolateral ligament, and anterior arm of the biceps femoris in knee trauma. Skeletal Radiol.

[22] Dietz GW, Wilcox DM, Montgomery JB (1986). Segond tibial condyle fracture: lateral capsular ligament avulsion. Radiology.

[23] Dodds AL, Halewood C, Gupte CM, Williams A, Amis AA (2014) The anterolateral ligament: Anatomy, length changes and association with the Segond fracture. Bone Jnt J 96-B(3):325–331. doi:10.1302/0301-620x.96b3.33033 10.1302/0301-620X.96B3.33033 24589786

[24] Ellera Gomes JL (1992). Medial patellofemoral ligament reconstruction for recurrent dislocation of the patella: a preliminary report. Arthrosc J Arthrosc Related Surg Off Publ Arthrosc Assoc N Am Int Arthrosc Assoc.

[25] Feagin JA (1988) The crucial ligaments: diagnosis and treatment of ligamentous injuries about the knee. Churchill Livingstone, New York

[26] Ferretti A, Monaco E, Fabbri M, Maestri B, De Carli A (2016). Prevalence and classification of injuries of anterolateral complex in acute anterior cruciate ligament tears. Arthroscopy.

[27] Hartigan DE, Carroll KW, Kosarek FJ, Piasecki DP, Fleischli JF, D’Alessandro DF (2016). Visibility of anterolateral ligament tears in anterior cruciate ligament-deficient knees with standard 1.5-Tesla magnetic resonance imaging. Arthrosc J Arthrosc Related Surg Off Publ Arthrosc Assoc N Am Int Arthrosc Assoc.

[28] Heckmann N, Sivasundaram L, Villacis D, Kleiner M, Yi A, White E, Rick Hatch GF (2016). Radiographic landmarks for identifying the anterolateral ligament of the knee. Arthrosc J Arthrosc Related Surg Off Publ Arthrosc Assoc N Am Int Arthrosc Assoc.

[29] Helito CP, Bonadio MB, Gobbi RG, da Mota EARF, Pecora JR, Camanho GL, Demange MK (2015). Combined intra- and extra-articular reconstruction of the anterior cruciate ligament: the reconstruction of the knee anterolateral ligament. Arthrosc Tech.

[30] Helito CP, Bonadio MB, Gobbi RG, da Mota EARF, Pecora JR, Camanho GL, Demange MK (2016). Is it safe to reconstruct the knee anterolateral ligament with a femoral tunnel? Frequency of lateral collateral ligament and popliteus tendon injury. Int Orthop.

[31] Helito CP, Bonadio MB, Soares TQ, da Mota e Albuquerque RF, Natalino RJ, Pecora JR, Camanho GL, Demange MK (2016). The meniscal insertion of the knee anterolateral ligament. Surg Radiol Anat SRA.

[32] Helito CP, Demange MK, Bonadio MB, Tirico LE, Gobbi RG, Pecora JR, Camanho GL (2013). Anatomy and histology of the knee anterolateral ligament. Orthop J Sports Med.

[33] Helito CP, Demange MK, Bonadio MB, Tirico LE, Gobbi RG, Pecora JR, Camanho GL (2014). Radiographic landmarks for locating the femoral origin and tibial insertion of the knee anterolateral ligament. Am J Sports Med.

[34] Helito CP, Helito PV, Costa HP, Bordalo-Rodrigues M, Pecora JR, Camanho GL, Demange MK (2014). MRI evaluation of the anterolateral ligament of the knee: assessment in routine 1.5-T scans. Skeletal Radiol.

[35] Helito CP, Helito PV, Costa HP, Demange MK, Bordalo-Rodrigues M (2016). Assessment of the anterolateral ligament of the knee by magnetic resonance imaging in acute injuries of the anterior cruciate ligament. Arthrosc J Arthrosc Related Surg Off Publ Arthrosc Assoc N Am Int Arthrosc Assoc.

[36] Hewison CE, Tran MN, Kaniki N, Remtulla A, Bryant D, Getgood AM (2015). Lateral extra-articular tenodesis reduces rotational laxity when combined with anterior cruciate ligament reconstruction: a systematic review of the literature. Arthrosc J Arthrosc Related Surg Off Publ Arthrosc Assoc N Am Int Arthrosc Assoc.

[37] Hughston JC, Andrews JR, Cross MJ, Moschi A (1976) Classification of knee ligament instabilities. Part II. The lateral compartment. J Bone Jnt Surg Am 58(2):173–179 1254620

[38] Ingham SJ, de Carvalho RT, Martins CA, Lertwanich P, Abdalla RJ, Smolinski P, Lovejoy CO, Fu FH (2015). Anterolateral ligament anatomy: a comparative anatomical study. Knee Surg Sports Traumatol Arthrosc Off J ESSKA.

[39] Irvine GB, Dias JJ, Finlay DB (1987). Segond fractures of the lateral tibial condyle: brief report. J Bone Jnt Surg Br.

[40] Kamath GV, Murphy T, Creighton RA, Viradia N, Taft TN, Spang JT (2014). Anterior cruciate ligament injury, return to play, and reinjury in the Elite Collegiate athlete: analysis of an NCAA Division I Cohort. Am J Sports Med.

[41] Kamath GV, Redfern JC, Burks RT (2010). Femoral radiographic landmarks for lateral collateral ligament reconstruction and repair: a new method of reference. Am J Sports Med.

[42] Kennedy MI, Claes S, Fuso FA, Williams BT, Goldsmith MT, Turnbull TL, Wijdicks CA, LaPrade RF (2015). The anterolateral ligament: an anatomic, radiographic, and biomechanical analysis. Am J Sports Med.

[43] Kernkamp WA, van de Velde SK, Bakker EW, van Arkel ER (2015). Anterolateral extra-articular soft tissue reconstruction in anterolateral rotatory instability of the knee. Arthrosc Tech.

[44] Kittl C, El-Daou H, Athwal KK, Gupte CM, Weiler A, Williams A, Amis AA (2016). The role of the anterolateral structures and the ACL in controlling laxity of the intact and ACL-deficient knee. Am J Sports Med.

[45] Kittl C, Halewood C, Stephen JM, Gupte CM, Weiler A, Williams A, Amis AA (2015). Length change patterns in the lateral extra-articular structures of the knee and related reconstructions. Am J Sports Med.

[46] Lemaire M (1967). Ruptures anciennes du ligament croisé antérieur. J Chir (Paris).

[47] Lind M, Menhert F, Pedersen AB (2012). Incidence and outcome after revision anterior cruciate ligament reconstruction: results from the Danish registry for knee ligament reconstructions. Am J Sports Med.

[48] Lohmander LS, Englund PM, Dahl LL, Roos EM (2007). The long-term consequence of anterior cruciate ligament and meniscus injuries: osteoarthritis. Am J Sports Med.

[49] Lutz C, Sonnery-Cottet B, Imbert P, Barbosa NC, Tuteja S, Jaeger JH (2016). combined anterior and anterolateral stabilization of the knee with the iliotibial band. Arthrosc Tech.

[50] Lutz C, Sonnery-Cottet B, Niglis L, Freychet B, Clavert P, Imbert P (2015). Behavior of the anterolateral structures of the knee during internal rotation. Orthop Traumatol Surg Res OTSR.

[51] Macchi V, Porzionato A, Morra A, Stecco C, Tortorella C, Menegolo M, Grignon B, De Caro R (2016). The anterolateral ligament of the knee: a radiologic and histotopographic study. Surg Radiol Anat.

[52] MacIntosh DL, Darby TA (1976) Lateral substitution reconstruction (abstract). J Bone Joint Surg 58(B):142

[53] Monaco E, Ferretti A, Labianca L, Maestri B, Speranza A, Kelly MJ, D’Arrigo C (2012). Navigated knee kinematics after cutting of the ACL and its secondary restraint. Knee Surg Sports Traumatol Arthrosc Off J ESSKA.

[54] Moyen BJ, Jenny JY, Mandrino AH, Lerat JL (1992). Comparison of reconstruction of the anterior cruciate ligament with and without a Kennedy ligament-augmentation device. A randomized, prospective study. J Bone Jnt Surg Am.

[55] Muller B, Hofbauer M, Rahnemai-Azar AA, Wolf M, Araki D, Hoshino Y, Araujo P, Debski RE, Irrgang JJ, Fu FH, Musahl V (2016). Development of computer tablet software for clinical quantification of lateral knee compartment translation during the pivot shift test. Comp Methods Biomech Biomed Eng.

[56] Muller U, Kruger-Franke M, Schmidt M, Rosemeyer B (2015). Predictive parameters for return to pre-injury level of sport 6 months following anterior cruciate ligament reconstruction surgery. Knee Surg Sports Traumatol Arthrosc Off J ESSKA.

[57] Müller W (1982) The knee: form, function and ligamentous reconstruction surgery. Springer, Berlin

[58] Muneta T, Koga H, Morito T, Yagishita K, Sekiya I (2006). A retrospective study of the midterm outcome of two-bundle anterior cruciate ligament reconstruction using quadrupled semitendinosus tendon in comparison with one-bundle reconstruction. Arthrosc J Arthrosc Related Surg Off Publ Arthrosc Assoc N Am Int Arthrosc Assoc.

[59] Musahl V, Hoshino Y, Ahlden M, Araujo P, Irrgang JJ, Zaffagnini S, Karlsson J, Fu FH (2012). The pivot shift: a global user guide. Knee Surg Sports Traumatol Arthrosc Off J ESSKA.

[60] Musahl V, Rahnemai-Azar AA, van Eck CF, Guenther D, Fu FH (2016). Anterolateral ligament of the knee, fact or fiction?. Knee Surg Sports Traumatol Arthrosc Off J ESSKA.

[61] Myer GD, Paterno MV, Ford KR, Quatman CE, Hewett TE (2006). Rehabilitation after anterior cruciate ligament reconstruction: criteria-based progression through the return-to-sport phase. J Orthop Sports Phys Ther.

[62] Nedeff DD, Bach BR (2001). Arthroscopic anterior cruciate ligament reconstruction using patellar tendon autografts: a comprehensive review of contemporary literature. Am J Knee Surg.

[63] Nitri M, Rasmussen MT, Williams BT, Moulton SG, Cruz RS, Dornan GJ, Goldsmith MT, LaPrade RF (2016). An In Vitro robotic assessment of the anterolateral ligament, part 2: anterolateral ligament reconstruction combined with anterior cruciate ligament reconstruction. Am J Sports Med.

[64] Norwood LA, Andrews JR, Meisterling RC, Glancy GL (1979). Acute anterolateral rotatory instability of the knee. J Bone Jnt Surg Am.

[65] Oshima T, Nakase J, Numata H, Takata Y, Tsuchiya H (2016). Ultrasonography imaging of the anterolateral ligament using real-time virtual sonography. Knee.

[66] Parsons EM, Gee AO, Spiekerman C, Cavanagh PR (2015). The biomechanical function of the anterolateral ligament of the knee. Am J Sports Med.

[67] Paterno MV, Rauh MJ, Schmitt LC, Ford KR, Hewett TE (2014). Incidence of Second ACL injuries 2 years after primary ACL reconstruction and return to sport. Am J Sports Med.

[68] Peccin MSG, M.;Parreira, P (2003) Princípios da reabilitação apos reconstrução do ligamento cruzado anterior. Lesões no esporte. Revinter

[69] Puddu GF, Mariani PP, Conteduca F (1987) Lesioni combinateanteriori acute. Il Ginocchio 6:303–306

[70] Pujol N, Blanchi MP, Chambat P (2007). The incidence of anterior cruciate ligament injuries among competitive Alpine skiers: a 25-year investigation. Am J Sports Med.

[71] Rasmussen MT, Nitri M, Williams BT, Moulton SG, Cruz RS, Dornan GJ, Goldsmith MT, LaPrade RF (2016). An In Vitro robotic assessment of the anterolateral ligament, part 1: secondary role of the anterolateral ligament in the setting of an anterior cruciate ligament injury. Am J Sports Med.

[72] Rezansoff AJ, Caterine S, Spencer L, Tran MN, Litchfield RB, Getgood AM (2015). Radiographic landmarks for surgical reconstruction of the anterolateral ligament of the knee. Knee Surg Sports Traumatol Arthrosc Off J ESSKA.

[73] Risberg MA, Lewek M, Snyder-Mackler L (2004). A systematic review of evidence for anterior cruciate ligament rehabilitation, how much and what type. Phys Ther Sport.

[74] Roessler PP, Schuttler KF, Heyse TJ, Wirtz DC, Efe T (2016). The anterolateral ligament (ALL) and its role in rotational extra-articular stability of the knee joint: a review of anatomy and surgical concepts. Arch Orthop Trauma Surg.

[75] Roth JH, Kennedy JC, Lockstadt H, McCallum CL, Cunning LA (1987). Intra-articular reconstruction of the anterior cruciate ligament with and without extra-articular supplementation by transfer of the biceps femoris tendon. J Bone Jnt Surg Am.

[76] Saiegh YA, Suero EM, Guenther D, Hawi N, Decker S, Krettek C, Citak M, Omar M (2015). Sectioning the anterolateral ligament did not increase tibiofemoral translation or rotation in an ACL-deficient cadaveric model. Knee Surg Sports Traumatol Arthrosc Off J ESSKA.

[77] Schottle PB, Schmeling A, Rosenstiel N, Weiler A (2007). Radiographic landmarks for femoral tunnel placement in medial patellofemoral ligament reconstruction. Am J Sports Med.

[78] Segond P (1879) Recherches cliniques et experimentales sur les epanchements sanguins du genou par entorse. Progres Medical 7:297–299, 319–321, 340–341

[79] Shaffer MA, Williams GN (2012) ACL rehabilitation. In: The Knee Joint. Surgical techniques and strategies. Springer, Paris, pp 269–287

[80] Shybut TB, Vega CE, Haddad J, Alexander JW, Gold JE, Noble PC, Lowe WR (2015). Effect of lateral meniscal root tear on the stability of the anterior cruciate ligament-deficient knee. Am J Sports Med.

[81] Siebold R, Takada T, Feil S, Dietrich C, Stinton SK, Branch TP (2016). Anatomical “C”-shaped double-bundle versus single-bundle anterior cruciate ligament reconstruction in pre-adolescent children with open growth plates. Knee Surg Sports Traumatol Arthrosc Off J ESSKA.

[82] Slette EL, Mikula JD, Schon JM, Marchetti DC, Kheir MM, Turnbull TL, LaPrade RF (2016). Biomechanical results of lateral extra-articular tenodesis procedures of the knee: a systematic review. Arthroscopy.

[83] Smith JO, Yasen SK, Lord B, Wilson AJ (2015). Combined anterolateral ligament and anatomic anterior cruciate ligament reconstruction of the knee. Knee Surg Sports Traumatol Arthrosc Off J ESSKA.

[84] Smith TO, Walker J, Russell N (2007). Outcomes of medial patellofemoral ligament reconstruction for patellar instability: a systematic review. Knee Surg Sports Traumatol Arthrosc Off J ESSKA.

[85] Song GY, Zhang H, Wang QQ, Zhang J, Li Y, Feng H (2016). Risk factors associated with grade 3 pivot shift after acute anterior cruciate ligament injuries. Am J Sports Med.

[86] Sonnery-Cottet B, Barbosa NC, Tuteja S, Daggett M, Kajetanek C, Thaunat M (2016). Minimally invasive anterolateral ligament reconstruction in the setting of anterior cruciate ligament injury. Arthrosc Tech.

[87] Sonnery-Cottet B, Lutz C, Daggett M, Dalmay F, Freychet B, Niglis L, Imbert P (2016). The involvement of the anterolateral ligament in rotational control of the knee. Am J Sports Med.

[88] Sonnery-Cottet B, Thaunat M, Freychet B, Pupim BH, Murphy CG, Claes S (2015). Outcome of a combined anterior cruciate ligament and anterolateral ligament reconstruction technique with a minimum 2-year follow-up. Am J Sports Med.

[89] Stijak L, Bumbasirevic M, Radonjic V, Kadija M, Puskas L, Milovanovic D, Filipovic B (2014). Anatomic description of the anterolateral ligament of the knee. Knee Surg Sports Traumatol Arthrosc.

[90] Stoehr AM, Wondrasch B, Fink C (2014) Rehabilitation and Return to Sports. Anterior Cruciate Ligament Reconstruction. A practical surgical guide. Springer

[91] Tanaka M, Vyas D, Moloney G, Bedi A, Pearle AD, Musahl V (2012). What does it take to have a high-grade pivot shift?. Knee Surg Sports Traumatol Arthrosc Off J ESSKA.

[92] Taneja AK, Miranda FC, Braga CA, Gill CM, Hartmann LG, Santos DC, Rosemberg LA (2015). MRI features of the anterolateral ligament of the knee. Skeletal Radiol.

[93] Tavlo M, Eljaja S, Jensen JT, Siersma VD, Krogsgaard MR (2015). The role of the anterolateral ligament in ACL insufficient and reconstructed knees on rotatory stability: a biomechanical study on human cadavers. Scand J Med Sci Sports.

[94] Terry GC, Norwood LA, Hughston JC, Caldwell KM (1993). How iliotibial tract injuries of the knee combine with acute anterior cruciate ligament tears to influence abnormal anterior tibial displacement. Am J Sports Med.

[95] Tiamklang T, Sumanont S, Foocharoen T, Laopaiboon M (2012). Double-bundle versus single-bundle reconstruction for anterior cruciate ligament rupture in adults. Cochrane Database Syst Rev.

[96] Trojani C, Beaufils P, Burdin G, Bussiere C, Chassaing V, Djian P, Dubrana F, Ehkirch FP, Franceschi JP, Hulet C, Jouve F, Potel JF, Sbihi A, Neyret P, Colombet P (2012). Revision ACL reconstruction: influence of a lateral tenodesis. Knee Surg Sports Traumatol Arthrosc Off J ESSKA.

[97] Trojani C, Sbihi A, Djian P, Potel JF, Hulet C, Jouve F, Bussiere C, Ehkirch FP, Burdin G, Dubrana F, Beaufils P, Franceschi JP, Chassaing V, Colombet P, Neyret P (2011). Causes for failure of ACL reconstruction and influence of meniscectomies after revision. Knee Surg Sports Traumatol Arthrosc.

[98] Van Dyck P, Clockaerts S, Vanhoenacker FM, Lambrecht V, Wouters K, De Smet E, Gielen JL, Parizel PM (2016). Anterolateral ligament abnormalities in patients with acute anterior cruciate ligament rupture are associated with lateral meniscal and osseous injuries. Eur Radiol.

[99] Van Dyck P, De Smet E, Lambrecht V, Heusdens CH, Van Glabbeek F, Vanhoenacker FM, Gielen JL, Parizel PM (2016). The anterolateral ligament of the knee: what the radiologist needs to know. Semin Musculoskelet Radiol.

[100] van Grinsven S, van Cingel RE, Holla CJ, van Loon CJ (2010). Evidence-based rehabilitation following anterior cruciate ligament reconstruction. Knee Surg Sports Traumatol Arthrosc Off J ESSKA.

[101] Vieira EL, Vieira EA, da Silva RT, Berlfein PA, Abdalla RJ, Cohen M (2007). An anatomic study of the iliotibial tract. Arthrosc J Arthrosc Related Surg Off Publ Arthrosc Assoc N Am Int Arthrosc Assoc.

[102] Vincent JP, Magnussen RA, Gezmez F, Uguen A, Jacobi M, Weppe F, Al-Saati MF, Lustig S, Demey G, Servien E, Neyret P (2012). The anterolateral ligament of the human knee: an anatomic and histologic study. Knee Surg Sports Traumatol Arthrosc Off J ESSKA.

[103] Wiggins AJ, Grandhi RK, Schneider DK, Stanfield D, Webster KE, Myer GD (2016). Risk of secondary injury in younger athletes after anterior cruciate ligament reconstruction: a systematic review and meta-analysis. Am J Sports Med.

[104] Williams GN, Chmielewski T, Rudolph K, Buchanan TS, Snyder-Mackler L (2001). Dynamic knee stability: current theory and implications for clinicians and scientists. J Orthop Sports Phys Ther.

[105] Woods GW, Stanley RF, Tullos HS (1979). Lateral capsular sign: X-ray clue to a significant knee instability. Am J Sports Med.

[106] Zaffagnini S, Lopomo N, Signorelli C, Marcheggiani Muccioli GM, Bonanzinga T, Grassi A, Visani A, Marcacci M (2013). Innovative technology for knee laxity evaluation: clinical applicability and reliability of inertial sensors for quantitative analysis of the pivot-shift test. Clin Sports Med.

